# Nutrition literacy and salt reduction measures: a mediating role of salt reduction intention

**DOI:** 10.3389/fnut.2025.1603835

**Published:** 2025-06-26

**Authors:** Xiaoting Hu, Yuhui Sun, Xi Tian, Huaqing Liu

**Affiliations:** School of Public Health, Bengbu Medical University, Bengbu, Anhui, China

**Keywords:** nutrition literacy, salt reduction measures, salt reduction intention, adults, mediation analysis

## Abstract

**Background:**

Excessive salt intake is a public health issue, posing considerable risks to individuals’ health. Various salt reduction strategies are being implemented globally. Nutrition literacy (NL) enables people to make well-informed food decisions that impact their well-being. Individual’s behavior is primarily shaped by their intentions, which, in turn, are influenced by their knowledge and attitudes regarding the action. The current study aims to estimate the relationships between NL and salt reduction measures and examine whether the intention to reduce salt intake mediates these relationships.

**Methods:**

This is a cross-sectional study conducted among adults in Bengbu, China. NL was assessed using a twelve-item short-form NL scale. Salt reduction measures and intention were identified through questionnaires. A binary logistic regression model was applied to calculate the odds ratio (*OR*) and 95% confidence interval (*CI*) for this association.

**Results:**

Overall, 50.7% of participants indicated implementing salt reduction measures, with 32.5% indicating a strong intention to do so. NL showed a significant positive association with the salt reduction intention (*OR* = 1.07, 95% *CI*: 1.06–1.09) and measures (*OR* = 1.06, 95% *CI*:1.04–1.07). This association was particularly evident in checking nutritional labels for sodium content (*OR* = 1.08, 95% *CI*: 1.05–1.10), using low-sodium salt (*OR* = 1.06, 95% *CI*: 1.04–1.08), and using salt-restriction tools (*OR* = 1.05, 95% *CI*: 1.03–1.07). And, the salt reduction intention (mediation effect ratio = 66.7%) mediated the relationship between NL and salt reduction measures.

**Conclusion:**

NL is positively associated with salt reduction measures, and salt reduction intention partially mediate their associations. These findings underscore the need for NL-targeted interventions or programs that enhance individuals’ intention to adopt salt reduction measures within the Chinese population.

## 1 Introduction

Salt, primarily composed of sodium, is universally used worldwide. Sodium is a critical electrolyte that affects fluid balance, immunometabolism, cellular functions, and muscle contraction (1–3). Nevertheless, the pervasive global consumption of salt has reached alarming levels. In 2017, the global average daily salt intake was 15 g/d (4). Excessive consumption of sodium has been related to numerous human health issues such as elevated blood pressure, stroke, cognitive impairment, and an increased burden of chronic kidney disease, all of which pose substantial public health challenges (5–7). Notably, excessive sodium consumption was identified as one of predominant risk factors of dietary-related mortality, directly accounting for 3.2 million deaths (8).

The WHO has advocated for a 30% decrease in the average population’s salt consumption by 2025 (9). Diverse strategies for salt reduction are being implemented globally. The United States Dietary Guidelines recommend ‘avoiding excessive sodium’ by adopting several measures such as cooking with minimal salt, adding little or no salt at the table, limiting the consumption of salty foods, and reading food labels (10). In Nigeria, there is a proposal to engage industries in reformulating their products to reduce sodium content, improving product labeling, and rolling out numerous educational initiatives across sectors to diminish sodium consumption (11). China has introduced food labeling and is offering low-sodium salt and salt restriction tools through community-based primary health care centers (12). Despite these efforts, recent studies have suggested that most populations across the globe exceed the recommended daily salt consumption (13–15).

Dietary behaviors are primarily influenced by social and cultural factors, as well as population characteristics such as age, education level, and income. However, modifying these aspects within a short term poses significant challenges (16). It is believed that knowledge and intention play a role in influencing the consumption of salt, moreover, these factors are regarded as modifiable mediators (17, 18). The Knowledge-Attitude-behavior (KAB) theory is a widely accepted framework for behavioral intervention. The KAB model suggests that exposure to new information can enhance knowledge acquisition, alter attitudes, and ultimately lead to the adoption of healthier dietary habits (19). Several studies suggest that knowledge and attitude could predict consumers’ salt-related behaviors (20, 21). Evidence from an intervention study showed that hypertensive patients achieved improvements in both knowledge and salt-related dietary behaviors following educational intervention (22). However, results have been inconsistent. Despite some individuals being aware of the health risks linked to excessive salt consumption, this knowledge does not always translate into a reduction in intake. For instance, Kolsom observed no significant association between knowledge toward salt intake and reduced consumption (23). Moreover, another cross-sectional survey reported that among participants who are aware of salt reduction interventions, less than half actively engaged in their implementation (24). Hence, it needs to identify key factors that impact salt reduction efforts for the development of tailored and effective interventions.

Nutrition literacy (NL) is a concept rooted in health literacy (25), referring to the extent to which individuals are equipped with the ability to obtain, process, and understand nutrition-related information, alongside the competencies necessary for informed nutritional decision-making (26). NL goes beyond merely acquiring knowledge about nutrition; it also encompasses application, assessing an individual’s capability to independently address nutrition-related problems (27). It empowers individuals to make correct health-influencing dietary choices and plays a pivotal role in shaping dietary quality and dietary behaviors (27, 28). From the standpoint of public health, NL could be a vital potential factor in promoting salt reduction strategies. To date, however, their relationship remains underexplored. Drawing on existing evidence, we propose the hypotheses that NL impacts salt reduction intention, which subsequently influences salt reduction measures. This study aims to comprehensively evaluate the associations between NL and salt reduction measures, as well as the potential mediating role of salt reduction intention among Chinese adults, providing a scientific foundation for developing strategies to enhance salt reduction measures in the prevention and control of related chronic diseases.

## 2 Materials and methods

### 2.1 Study design and participants

The cross-sectional survey, conducted between May and July 2023, aimed to investigate the association between NL and health in individuals aged 18 and older in Bengbu, China. A stratified multi-stage randomized sampling method was applied to select participants, ensuring a representative sample. More details have been described in previous research (29). A structured questionnaire was used to gather data on demographic characteristics, lifestyle, NL, salt reduction measures, and salt reduction intention. All participants voluntarily took part in the study and provided written informed consent. Ethical approval for the study was granted by the Ethical Review Commission of Bengbu Medical University Initially (2021-099).

To ensure the integrity of the questionnaire survey, trained investigators conducted one-on-one interviews following standardized protocols. Additionally, inspectors review and correct errors in the on-site questionnaires. A total of 2,279 respondents initially completed the interview. Respondents with missing data, contradictory answers, and clear repetitions (*n* = 17) were excluded. As a result, 2,262 adult individuals were finally included in this study.

### 2.2 NL assessment

A 12-item short-form NL assessment tool was utilized to assess participants’ NL (30). This tool provides a comprehensive evaluation of NL across six dimensions: knowledge, understanding, obtaining, applying, interactive and critical skills, and exhibits acceptable validity in Chinese adults (30–32). The NL scale employs a 5-point Likert scale for each item, and the total NL score is calculated based on the 12 items, with higher scores indicating higher levels of NL.

### 2.3 Salt reduction intention and measures

Salt reduction measures were determined by the question: “Do you adopt any salt reduction measures?” Response included “yes” and “no.” Those who responded affirmatively were subsequently question about the specific methods they employ for salt reduction, including “checking nutritional labels for sodium content,” “eat less salt-rich foods,” “cooking with less salt,” “no extra salt at the table,” “using low sodium salt,” and “using salt-restriction tools.” Salt reduction intention was determined by responses to the following question: “How strong is your salt reduction intention?” The possible responses were: no intention at all, no intention, neutral intention, strong intention, very strong intention.

### 2.4 Other variables

We accounted for potential confounding effects by adjusting for the following covariates: age/years (18–44, 45–64 or 65 and above), gender (male or female), residence (urban or rural), marital status (married or other), BMI/kg/m^2^ (<18.5, 18.5–24, 24–28, ≥28), educational attainment (primary and below, junior high school and above), occupational status (farmers, separated/retired staff, or others), monthly salary/RMB (< 3,000 or ≥3,000), drinking (yes or no), smoking (yes or no), exercise (yes or no), and chronic diseases (yes or no).

### 2.5 Statistical analysis

The sample size and the percentage of respondents were employed to outline the participants’ characteristics and salt reduction measures, and the difference was verified by the χ^2^ test. As a continuous variable, NL is presented as means ± standard deviations, and the Kruskal-Wallis H test was used to evaluate the significance of differences in NL between two groups: ‘yes’ and ‘no’ for salt reduction measures. After controlling for age, gender, occupational status, marital status, BMI, educational attainment, residence, monthly salary, smoking, drinking, exercise, and chronic diseases, the association between NL and salt reduction measures was evaluated through binary logistic regression model by computing the odds ratio (*OR*) and its 95% confidence interval (*CI*).

**Figure 1:**
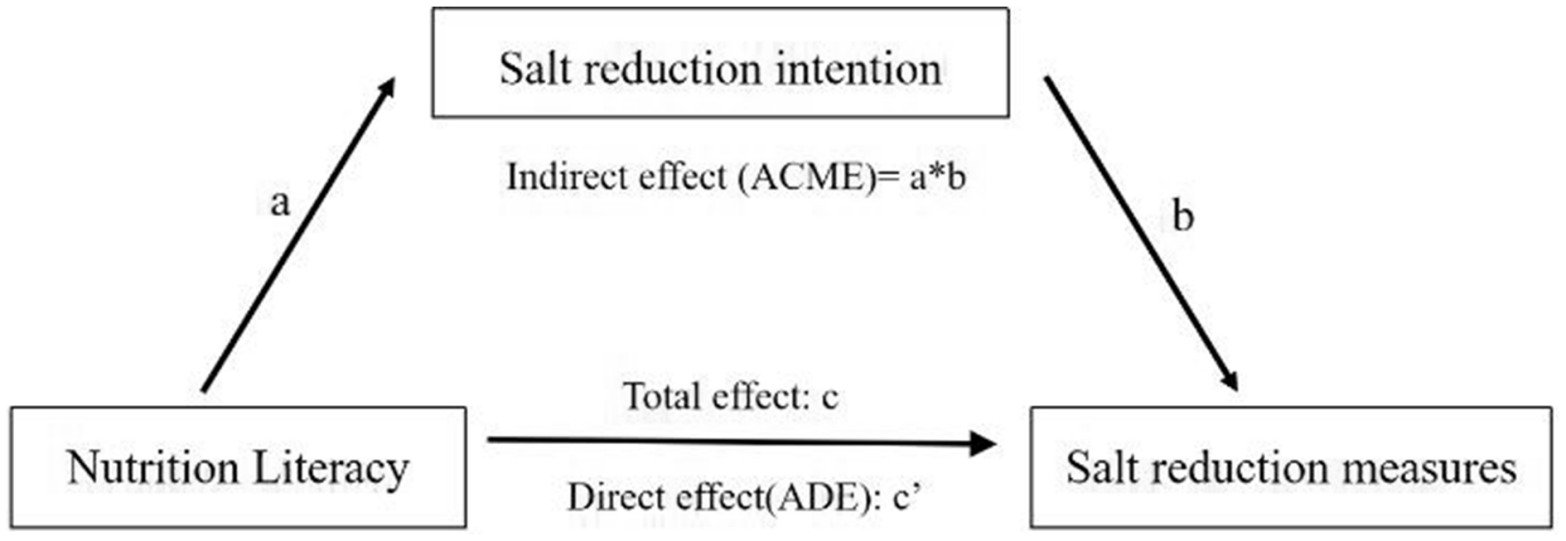
Model of salt reduction intention mediating the association between NL and salt reduction measures. a = direct effect of independent variable (IV) on mediator (M). b = direct effect of mediator on dependent variable (DV). c = total effect of IV on DV. c’ = direct effect of IV on DV.

A model-based causal mediation analysis was conducted using a hypothetical model to assess the mediation of the effect of salt reduction intention on the association between NL and salt reduction measures (Figure 1). The average direct effect (ADE) quantified the impact of NL on salt reduction measures, while the average causal mediation effect (ACME) represented the effect mediated by salt reduction intention. The mediation effect ratio mediated by intention was estimated as ACME/ [ADE + ACME] *100%.

The research employed SPSS 23.0 for statistical data processing, with mediation analysis performed using the ‘mediation’ package in R 4.4.2, based on 1,000 bootstrap repeated samples, and a two-sided significance level of *p* < 0.05.

## 3 Results

### 3.1 Participant characteristics

The baseline characteristics of the participants are shown in Table 1. Of the total sample of 2,262 individuals, 50.7% of participants had adopted salt reduction measures, 32.5% had a strong intention to adopt them, and 5.5% had a very strong intention. The percentages of participants aged 18–44, 45–64, and 65 years and above were 17.1, 30.9, and 52.0%, respectively. 61.5% of participants were female, 48.5% had completed no higher than primary school, 77.9% were married, and 31.2% were farmers, 54.2% lived in rural areas, and 40.5% had normal BMI. Most participants (71.0%) earned a monthly salary below 3,000 RMB. In addition, 72.7% of participants did not smoke, 64.0% of participants did not drink, 29.9% of participants did not engage in regular physical exercise, and 71.4% of participants had one or more chronic diseases.

**Table 1:** Salt reduction measures according to participants’ characteristics.

Variables	*N* (%)	Salt reduction measures *n* (%)	*χ* ^2^	*p*
Total	2,262 (100.0)	1,146 (50.7)		
Age group (years)			1.68	0.433
18–44	376 (17.1)	181 (16.2)		
45–64	679 (30.9)	355 (31.9)		
65–	1,143 (52.0)	578 (51.9)		
Gender			4.22	0.040
Male	872 (38.5)	418 (36.5)		
Female	1,390 (61.5)	728 (63.5)		
Residence			21.66	<0.001
Urban	1,036 (45.8)	580 (50.6)		
Rural	1,226 (54.2)	566 (49.4)		
BMI (kg/m^2^)			0.40	0.941
< 18.5	60 (2.7)	28 (2.5)		
18.5–23.9	911 (40.5)	461 (40.4)		
24–27.9	889 (39.5)	452 (39.6)		
≥28	392 (17.4)	199 (17.5)		
Educational attainment			24.09	<0.001
Primary and below	1,096 (48.5)	497 (43.4)		
Junior high school and above	1,162 (51.5)	647 (56.6)		
Marital status			10.07	0.002
Married	1,762 (77.9)	924 (80.6)		
Others	500 (22.1)	222 (19.4)		
Occupational status			13.68	0.001
Farmers	704 (31.2)	339 (29.7)		
Separated/retired staff	734 (32.6)	413 (36.2)		
Others	816 (36.2)	390 (34.2)		
Monthly salary			7.07	0.008
<3,000 RMB	1,591 (71.0)	779 (68.5)		
≥3,000 RMB	651 (29.0)	359 (31.5)		
Smoking			4.35	0.037
No	1,644 (72.7)	855 (74.6)		
Yes	618 (27.3)	291 (25.4)		
Drinking			5.35	0.021
No	1,448 (64.0)	760 (66.3)		
Yes	814 (36.0)	386 (33.7)		
Exercise			25.08	<0.001
No	676 (29.9)	288 (25.2)		
Yes	1,582 (70.1)	856 (74.8)		
Chronic diseases			2.03	0.155
No	648 (28.6)	313 (27.3)		
Yes	1,614 (71.4)	833 (72.7)		
Salt reduction intention			925.86	<0.001
No intention at all	295 (13.1)	29 (2.5)		
No intention	332 (14.7)	25 (2.2)		
Neutral intention	770 (34.1)	337 (29.5)		
Strong intention	734 (32.5)	633 (55.4)		
Very strong intention	125 (5.5)	118 (10.3)		

The univariate analysis revealed significant differences in salt reduction measures based on factors such as gender, occupational status, marital status, educational attainment, residence, monthly salary, smoking, drinking, exercise, and salt reduction intention (Table 1). However, no significant variation in salt reduction measures was observed across different age groups, BMI categories, or chronic diseases.

### 3.2 Specific salt reduction measures and their combinations

**Figure 2:**
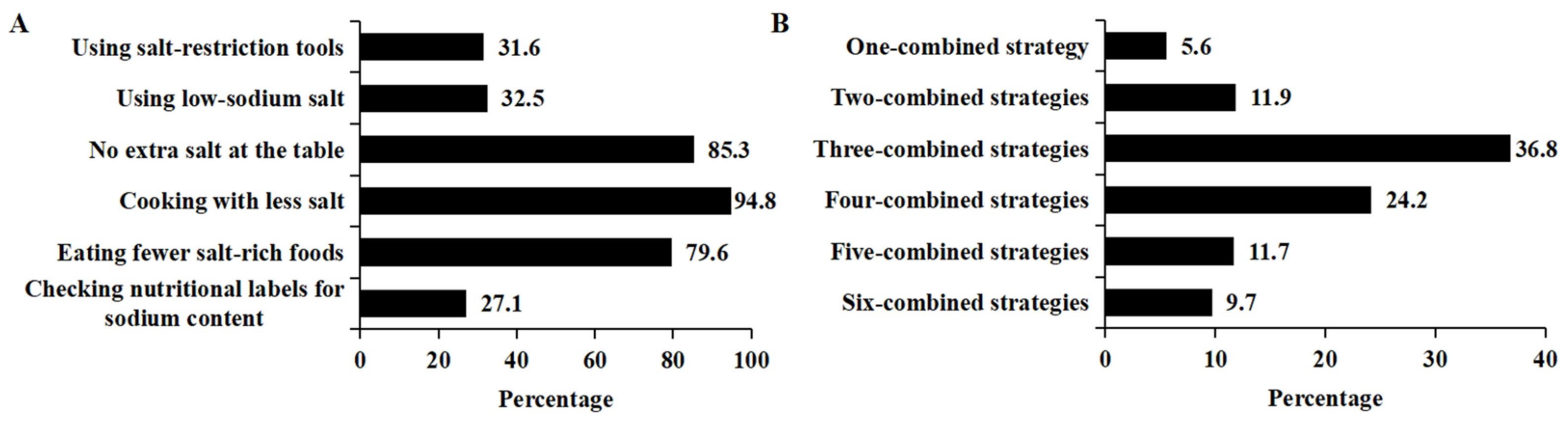
Specific salt reduction measures and their combinations. **(A)** The percentage of specific salt reduction measures. **(B)** The percentage of salt reduction measures’ combinations.

As shown in Figure 2A, 50.7% (1,146) of participants reported adopting salt reduction measures. Among the salt reduction measures, 31.6% used salt-restriction tools, 32.5% used low-sodium salt, 85.3% avoided adding extra salt at the table, 94.8% used less salt when cooking, 79.6% ate less salty foods, and 27.1% read the sodium content in the nutrition label when purchasing processed foods.

Among participants who adopted salt reduction measures, 82.4% of the respondents reported implementing three or more strategies concurrently. About a third (36.8%) of them reported adopting three different strategies concurrently (Figure 2B). Nearly one-tenth (9.7%) reported using six-combined strategies.

### 3.3 Nutrition literacy level according to salt reduction intention and measures

Table 2 showed the NL level according to salt reduction intention and measures. A significantly higher mean score of NL was observed in individuals with salt reduction measures or with very strong salt reduction intention (*p* < 0.05). This trend was also found on all six dimensions.

**Table 2:** The level of nutrition literacy by salt reduction intention and measures.

Variables	Salt reduction measures			Salt reduction intention					
	None	Yes	*H*	No intention at all	No intention	Neutral intention	Strong intention	Very strong intention	*H*
*Nutrition Literacy*	35.81 ± 8.32	39.42 ± 8.34	−10.24^***^	34.77 ± 10.32	34.81 ± 7.55	37.90 ± 7.59	38.87 ± 7.90	42.99 ± 10.52	120.63^***^
Knowledge	7.60 ± 1.59	8.14 ± 1.41	−8.36^***^	7.65 ± 1.98	7.32 ± 1.50	7.75 ± 1.39	8.14 ± 1.34	9.07 ± 3.04	164.84^***^
Understanding	5.79 ± 2.19	6.25 ± 2.24	−6.01^***^	5.72 ± 2.62	5.48 ± 1.97	6.23 ± 1.99	6.13 ± 2.16	7.10 ± 3.04	59.81^***^
Obtaining skills	5.20 ± 2.03	5.79 ± 2.16	−6.53^***^	4.72 ± 2.46	5.17 ± 1.87	5.70 ± 1.85	5.64 ± 2.07	6.03 ± 2.96	68.41^***^
Applying skills	4.83 ± 1.84	5.52 ± 1.98	−8.67^***^	4.45 ± 2.10	4.66 ± 1.68	5.40 ± 1.78	5.44 ± 1.96	5.42 ± 2.37	98.38^***^
Interactive skills	6.53 ± 1.72	7.17 ± 1.71	−9.31^***^	6.41 ± 2.28	6.51 ± 1.54	6.68 ± 1.56	7.16 ± 1.55	8.04 ± 2.10	127.31^***^
Critical skills	5.86 ± 1.77	6.45 ± 1.81	−7.78^***^	5.82 ± 2.22	6.13 ± 1.59	6.35 ± 1.71	7.34 ± 2.32	6.16 ± 1.81	86.65^***^

^*^p < 0.05, ^**^p < 0.01, ^***^p < 0.001.

### 3.4 Association of nutrition literacy with salt reduction intention and measures

As presented in Table 3, after controlling for age group, gender, occupation, marital status, BMI, educational attainment, residence, monthly salary, smoking, drinking, exercise and chronic diseases, NL was significantly and positively linked to salt reduction measures in adults (*OR* = 1.06, 95% *CI*: 1.04–1.07). This positive relationship was present in the dimension of knowledge (*OR* = 1.26, 95% *CI*: 1.18–1.34), understanding (*OR* = 1.10, 95% *CI*: 1.05–1.16), obtaining skills (*OR* = 1.13, 95% *CI*: 1.07–1.19), applying skills (*OR* = 1.18, 95% *CI*: 1.12–1.25), interactive skills (*OR* = 1.20; 95% *CI*: 1.14–1.27) and critical skills (*OR* = 1.18, 95% *CI*: 1.12–1.24). NL was also associated with salt reduction intention (*OR* = 1.07, 95% *CI*: 1.06–1.09); and, this association was observed in the six dimensions of NL.

**Table 3:** Association of nutrition literacy with salt reduction intention and measures.

Variables	Salt reduction measures	Specific measures for salt reduction (*n* = 1,146)						Salt reduction intention
		Checking nutritional labels for sodium content (*n* = 311)	Eating less salt-rich foods (*n* = 912)	Cooking with less salt (*n* = 1,086)	No extra salt at the table (*n* = 978)	Using low-sodium salt (*n* = 373)	Using salt-restriction tools (*n* = 362)	
	*OR* (95% *CI*)	*OR* (95% *CI*)	*OR* (95% *CI*)	*OR* (95% *CI*)	*OR* (95% *CI*)	*OR* (95% *CI*)	*OR* (95% *CI*)	*OR* (95% *CI*)
*Nutritional literacy*	1.06 (1.04,1.07)^***^	1.08 (1.05,1.10)^***^	1.02 (0.99,1.04)	0.99 (0.95,1.03)	1.01 (0.99,1.04)	1.06 (1.04,1.08)^***^	1.05 (1.03,1.07)^***^	1.07 (1.06,1.09)^***^
Knowledge	1.26 (1.18,1.34)^***^	1.17 (1.04,1.30)^**^	1.12 (1.01,1.25)^*^	1.13 (0.94,1.36)	1.10 (0.97,1.25)	1.18 (1.06,1.31)^**^	1.18 (1.06,1.30)^**^	1.35 (1.28,1.43)^***^
Understanding	1.10 (1.05,1.16)^***^	1.19 (1.10,1.28)^***^	0.98 (0.91,1.06)	0.87 (0.75,1.00)^*^	0.98 (0.90,1.07)	1.16 (1.08,1.25)^***^	1.08 (1.01,1.16)^*^	1.14 (1.09,1.19)^***^
Obtaining skills	1.13 (1.07,1.19)^***^	1.23 (1.14,1.34)^***^	0.99 (0.91,1.08)	0.87 (0.756,1.00)	1.03 (0.93,1.13)	1.12 (1.04,1.21)^**^	1.10 (1.02,1.18)^*^	1.17 (1.12,1.23)^***^
Applying skills	1.18 (1.12,1.25)^***^	1.48 (1.35,1.61)^**^	1.01 (0.92,1.10)	0.93 (0.80,1.09)	0.95 (0.86,1.05)	1.24 (1.14,1.33)^***^	1.19 (1.10,1.28)^***^	1.19 (1.13,1.24)^***^
Interactive skills	1.20 (1.14,1.27)^***^	0.98 (0.90,1.07)	1.17 (1.07,1.28)^***^	1.20 (1.02,1.40)^*^	1.12 (1.01,1.24)^*^	1.07 (0.98,1.16)	1.05 (0.97,1.14)	1.27 (1.20,1.33)^***^
Critical skills	1.18 (1.12,1.24)^***^	1.23 (1.13,1.35)^***^	1.05 (0.96,1.15)	0.98 (0.83,1.14)	1.10 (0.99,1.22)	1.24 (1.14,1.34)^***^	1.21 (1.12,1.32)^***^	1.23 (1.17,1.23)^***^

^*^p < 0.05, ^**^p < 0.01, ^***^p < 0.001. OR represents the odds ratio, 95% CI represents 95% confidence intervals. The OR (95% CI) was calculated using binary logistic regression when adjusting for age group, gender, occupational status, marital status, BMI, educational attainment, residence, monthly salary (RMB), smoking (yes/no), drinking (yes/no), exercise (yes/no), and chronic diseases (yes/no).

Significant associations were also found between NL and checking nutritional labels for sodium content (*OR* = 1.08, 95% *CI*: 1.05–1.10), low-sodium salt use (*OR* = 1.06, 95% *CI*: 1.04–1.08), and salt-restriction tools use (*OR* = 1.05, 95% *CI*: 1.03–1.07). This relationship was also observed across knowledge, understanding, obtaining, applying, and critical skills. Although interactive skills were not significantly associated with these salt reduction measures, it was significantly associated with eating less salt-rich foods (*OR* = 1.17, 95% *CI*: 1.07–1.28), cooking with less salt (*OR* = 1.20, 95% *CI*: 1.02–1.40), and no extra salt at the table (*OR* = 1.12, 95% *CI*: 1.01–1.24).

### 3.5 Mediation analysis of salt reduction intention between NL and salt reduction measures

Our mediation analyses indicated that salt reduction intention explains the partial association between NL and salt reduction measures. To be specific, for NL and salt reduction measures, the mediating effect estimate (mediation effect ratio) was 0.004 (66.7%) for salt reduction intention. Further intermediary role analysis showed that salt reduction intention partially mediated the relationship between knowledge, applying skills, interactive, critical skills and salt reduction measures, with mediation effect ratio of 71.9, 61.1, 67.6, and 67.6%, respectively (Table 4). Additionally, the effects of understanding NL and obtaining skills on salt reduction measures were fully mediated by salt reduction intention.

**Table 4:** Mediation analysis of salt reduction intention between nutrition literacy and salt reduction measures.

Variables	Indirect effect (ACME) (a*b)		Direct effect (ADE) (c’)		Total effect (c)		Mediation effect ratio (%)
	Estimate	95% *CI*	Estimate	95% *CI*	Estimate	95% *CI*	
*Nutritional literacy*	0.004	(0.003,0.010)^***^	0.002	(0.000,0.001)^***^	0.006	(0.005,0.010)^***^	66.7
Knowledge	0.023	(0.017,0.030)^***^	0.009	(0.001,0.010)^*^	0.031	(0.029,0.030)^***^	71.9
Understanding	0.016	(0.009,0.020)^***^	0.009	(−0.001,0.020)	0.024	(0.014,0.030)^***^	64.0
Obtaining skills	0.020	(0.013,0.030)^***^	0.009	(−0.001,0.020)	0.030	(0.018,0.040)^***^	69.0
Applying skills	0.022	(0.016,0.030)^***^	0.014	(0.005,0.020)^**^	0.036	(0.027,0.040)^***^	61.1
Interactive skills	0.023	(0.017,0.030)^***^	0.011	(0.002,0.020)^*^	0.034	(0.026,0.040)^***^	67.6
Critical skills	0.023	(0.016,0.030)^***^	0.011	(0.001,0.020)^*^	0.034	(0.024,0.040)^***^	67.6

^*^p < 0.05, ^**^p < 0.01, ^***^p < 0.001. 95% CI represents 95% confidence intervals, mediation effect ratios = ACME/ [ADE + ACME] * 100%.

### 3.6 Subgroup analysis stratified by chronic diseases

**Figure 3:**
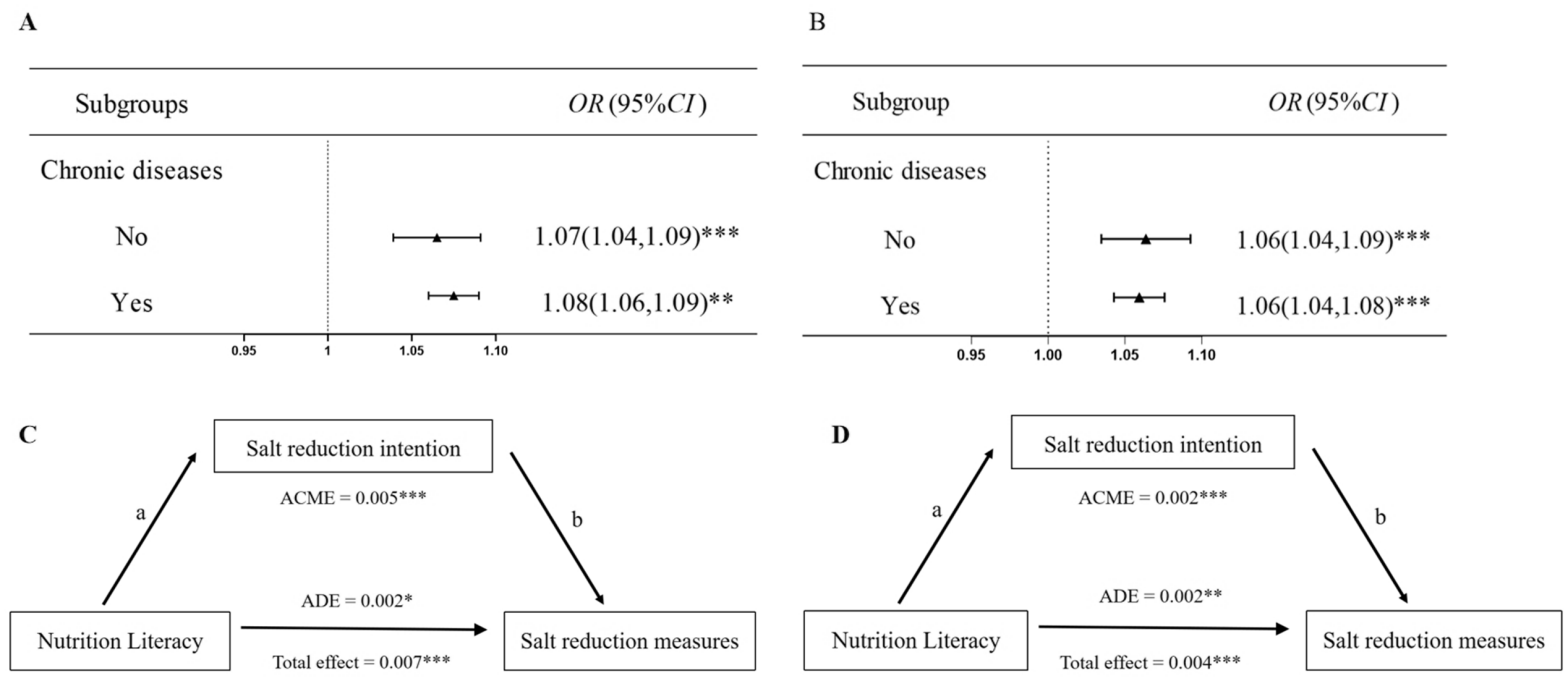
Subgroup analysis stratified by chronic diseases. **(A)** Association of nutrition literacy with salt reduction intention. **(B)** Association of nutrition literacy with salt reduction measures. **(C)** Mediating effect in populations without chronic diseases. **(D)** Mediating effect in populations with chronic diseases. a = direct effect of independent variable (IV) on mediator (M). b = direct effect of mediator on dependent variable (DV).^*^p < 0.05, ^**^p < 0.01, ^***^p < 0.001.

As illustrated in Figure 3A, a positive association was observed between NL and salt reduction intention both in people with chronic disease (*OR* = 1.08, 95% *CI* = 1.06 to 1.09) and in those without chronic disease (*OR* = 1.07, 95% *CI* = 1.04 to 1.09). This association also applied to NL and salt reduction measures (Figure 3B). Further analysis of the intermediary role showed that in the population with chronic disease, salt reduction intention partially explained the association between NL and salt reduction measures (Figure 3C). Similarly, a similar phenomenon was observed in the population without chronic disease (Figure 3D).

## 4 Discussion

It is the first study to explore the association of NL with salt reduction intention and measures. Findings showed that only half of adults had adopted salt reduction measures, with approximately one third having a strong intention to reduce salt intake. This evidence indicated that the awareness of the necessity to reduce salt intake remains relatively low. Therefore, there is a pressing need to bolster existing salt reduction measures. NL was positively associated with salt reduction measures; moreover, the intention to reduce salt intake partially mediates this association among Chinese adults, suggesting that interventions focused on NL could bolster the intention to reduce salt intake and encourage the implementation of salt reduction measures.

This study found that 94.8% of participants who implemented salt reduction measures adopted the practice of “cooking with less salt,” the most prevalent among all practices. Conversely, only 27.1% checked nutritional labels for sodium content, suggesting a lack of awareness about the importance of this practice when purchasing processed foods. In China, unlike developed countries (33), the primary source of sodium intake is salt added when cooking (12) rather than from processed foods. The utilization rates of “salt-restriction tools” and “low-sodium salt” were found to be relatively low at 31.6 and 32.5%, respectively. Notably, 36.8% of individuals who adopted salt reduction measures implemented three different strategies concurrently. These findings suggest the need for enhanced promotion of salt reduction measures, especially emphasizing the importance of “checking nutritional labels for sodium content,” “using low-sodium salt,” and “using salt-restriction tools.” Adopting a combination of these measures could potentially improve the overall effectiveness of reducing salt intake.

A significant positive relationship between NL and salt reduction measures was observed in our study, indicating that NL, beyond mere theoretical knowledge, plays a crucial role in translating that knowledge into practice (28). Adequate NL is conducive to fostering nutritional cognition, encouraging the practice of nutritional skills, and promoting healthier eating behaviors (34, 35). Specifically, individuals with higher NL (encompassing knowledge, understanding, obtaining skills, and applying skills) were more inclined to adopt beneficial salt-reduction practices such as checking nutritional labels for sodium content, using low-sodium salt, and employing salt-restriction tools. However, the analysis revealed no significant correlation between NL and eating less salt-rich foods, cooking with less salt, or not adding extra salt at the table. This could be attributed to the Chinese government’s ongoing efforts to elevate public consciousness about the detrimental effects of excessive consumption of salt, the recommended maximum salt consumption, and various salt reduction measures (12). The high adoption rate of these basic measures suggests widespread acceptance and ease of implementation in daily life, factors that may not be significantly influenced by NL. Furthermore, critical skills demonstrated a positive correlation with practices like checking nutritional labels for sodium content, using low-sodium salt, and utilizing salt-restriction tools. These skills enhance an individual’s analytical capabilities and understanding of measures, thereby facilitating the adoption of informed, health-conscious actions (31, 36). Interestingly, a relationship was observed between interactive skills and measures such as eating less salty foods, cooking with less salt, and not adding extra salt at the table. Previous research has suggested that interactive skills positively influence improved eating habits (36), with interactions within the family setting appearing to be particularly effective in regulating salt intake behaviors (23). These findings highlight the significance of increasing NL in public health salt reduction interventions. Furthermore, public health interventions that focus on the improvement of nutrition literacy may assist individuals in converting their knowledge into relevant healthy dietary habits—ultimately leading to effective promotion and implementation of salt reduction.

Our findings reported a significant positive relationship between NL levels and salt reduction intention among adults. This suggests that an individual who is of high NL may have a strong inclination to reduce salt consumption. Consistent with previous studies, diet-related knowledge, attitudes, and behaviors play a pivotal role in promoting healthy diet at an individual level (37). Furthermore, the study revealed that the intention to reduce salt intake partially mediates the relationship between NL and actual salt reduction measures, indicating that this intention can elucidate and impact the effect of NL on such measures. These findings supported the KAB theoretical model. The KAB model holds that dietary behavioral changes is the result of three successive processes: knowledge acquisition, belief generation, and behavior formation (38). The core of this theory is the individual’s knowledge and attitudes toward the action, which primarily shape their intention, thus affecting their behavior (19, 37, 39). Previous research has consistently shown that an individual who is of high NL is more likely to have great health awareness and is more motivated to adopt nutritionally appropriate behaviors (28, 40, 41). Notably, participants in this study who indicated an intention to reduce salt intake were more likely to demonstrate optimal salt control behavior (17). Moreover, the study underscores the significance of salt reduction intention in various aspects of NL that influence salt reduction measures.

The relationship between understanding, obtaining skills and implementing salt reduction measures was found to be fully mediated by salt reduction intention. Intention is regarded as a fundamental prerequisite for both adopting and performing a behavior (18). Thus, the findings suggest that even if an adult has achieved a high level of understanding and obtaining skills, they may not implement appropriate salt reduction measures without a sufficient intention to do so. In other words, salt reduction intention serves not only as an external motivator but also as a powerful mediating factor that strengthens the linking of understanding, obtaining skill with the implementation of salt reduction measures. To encourage salt reduction practices among adults, it is necessary to implement interventions that focus on enhancing NL and fostering a strong intention to reduce salt intake. Additionally, there is a pressing need to underscore the significance of holistic interventions that address both NL and salt reduction intentions comprehensively (18).

The present study pioneers an assessment of the relationship of NL with salt reduction intention and measures, establishing a pathway from NL to salt reduction intention and subsequently to salt reduction measures. However, our study has some limitations. First, it is a cross-sectional study, therefore, relationships between NL and salt reduction intention and measures cannot be used to make firm inferences regarding causality. Secondly, the evaluation of salt reduction awareness and related measures was dependent on subjective questioning. As such, participants may have overestimated their own choices, a tendency that is susceptible to inherent recall and reporting biases. Future research should consider employing objective measurements to mitigate these biases. Finally, this study did not adjust for family history of diseases, as no such data was collected. It could potentially affect the results.

## 5 Conclusion

This study highlighted a positive correlation between NL and the adoption of salt reduction measures, with this trend consistently observed across six further domains of NL. As NL increases, so too does the intention to reduce salt intake, partially mediating the relationship between NL and salt reduction actions. Specifically, NL plays a pivotal role in promoting behaviors such as checking nutritional labels for sodium content, using low-sodium salt, and utilizing salt-restriction tools. Interpersonal skills are crucial for implementing interventions aimed at reducing the consumption of salt-rich foods, cooking with less salt, and avoiding the addition of extra salt at the table. Findings underscore the need for NL-targeted interventions that enhance individuals’ intention to adopt salt reduction measures within the Chinese population.

## Data Availability

The raw data supporting the conclusions of this article will be made available by the authors, without undue reservation.
